# Fermentation as a Lever to Enhance Legume Proteins: From Antinutritional Factors to Improved Quality

**DOI:** 10.3390/molecules31173104

**Published:** 2026-09-04

**Authors:** Slim Smaoui, Abeera Moin, Haifa Chtourou, Olfa Ben Braïek, Mariam Fourati, Theodoros Varzakas

**Affiliations:** 1 Laboratory of Microbial and Enzymes Biotechnology and Biomolecules (LMEBB), Center of Biotechnology of Sfax (CBS), University of Sfax, Sfax 3029, Tunisia; mariamfourati@gmail.com; 2 Department of Food Science and Technology, University of Karachi, Karachi 75270, Pakistan; abeeramoin@uok.edu.pk; 3 Department of Process Engineering, Higher Institute of Technological Studies of Sfax (ISET Sfax), University of Sfax, Sfax 3029, Tunisia; haifach@yahoo.fr; 4 Laboratory of Enzyme Engineering and Microbiology, Engineering National School of Sfax (ENIS), University of Sfax, Sfax 3038, Tunisia; 5 Laboratory of Transmissible Diseases and Biologically Active Substances (LR99ES27), Faculty of Pharmacy of Monastir, University of Monastir, Monastir 5000, Tunisia; olfa_bbraiek@yahoo.fr; 6 Faculty of Sciences and Techniques of Sidi Bouzid, University of Kairouan, Kairouan 9100, Tunisia; 7 Department of Food Science and Technology, University of the Peloponnese, Antikalamos Messinia, 24100 Kalamata, Greece

**Keywords:** legume proteins, fermentation, antinutritional factors, protein digestibility, bioavailability, bioactive peptides, functional properties

## Abstract

Owing to their high nutritional value, functional proteins, and low environmental impact, legume proteins have emerged as sustainable alternatives to animal proteins. However, their broader utilization is constrained by antinutritional factors (ANFs), which can adversely affect protein digestibility, mineral bioavailability, and techno-functional features. Fermentation has appeared as a promising approach to overcoming these limitations. Through microbial and enzymatic activities, fermentation can reduce ANFs, modify protein structures, and promote proteolysis, generating peptides and free amino acids that may improve digestibility, bioavailability, and functional properties. This review critically explores the effects of fermentation on legume proteins, concentrating on ANF reduction, protein degradation and structural modification, proteolysis, and their consequences for nutritional and techno-functional properties. Particular attention is given to protein hydrolysis and peptide generation, as well as the limitations and potential trade-offs of fermentation, including extreme proteolysis, loss of desirable functional properties, unwanted sensory changes, variability among strains and substrates, and prolonged processing. The review also discusses the incorporation of fermented legume proteins into relevant food systems, with emphasis on their functional performance and application potential. Finally, current knowledge gaps and future research priorities are highlighted to support the development of controlled fermentation strategies for nutritionally improved, functionally tailored, and industrially feasible legume protein ingredients.

## 1. Introduction

Rich in protein and delivering exceptional nutritional quality, functional versatility, and a low environmental footprint, legume proteins have become leading sustainable alternatives to animal proteins [1]. In spite of their extraordinary nutritional potential, the wide application of legume proteins remains limited by the existence of antinutritional factors (ANFs). Obviously, legumes contain phytates, tannins, protease inhibitors, lectins, and other bioactive compounds that damage protein digestibility, decrease mineral bioavailability, and limit nutrient utilization, thus reducing their functional performance and application in food systems [2,3]. In terms of functionality, these compounds may form networks with proteins and minerals, decrease enzymatic accessibility, and limit the nutritional quality of legume-based foods [4]. In order to enhance legume protein quality, several physical, chemical, and enzymatic methods have been established. Nevertheless, biological approaches, particularly fermentation, have received cumulative interest as maintainable strategies for promoting the nutritional value and potential food application of legumes [5]. Fermentation includes the metabolic activity of microorganisms, which generate a wide range of enzymatic processes which can modify legume constituents, such as phytate degradation [6,7,8], proteases [9], and tannin [10]. These enzymatic activities contribute to the reduction of antinutritional factors, improvements in protein accessibility, and generation of bioactive compounds [11].

Outside ANF reduction, fermentation produces complex biochemical transformations in legume proteins. In fact, microbial proteolysis induces protein hydrolysis, subsequent to the release of peptides and free amino acids that may recover digestibility and generate bioactive peptides [12,13]. Additionally, fermentation can adapt protein structure, molecular interactions, and techno-functional characteristics—such as solubility, emulsification, foaming, and gelation features—increasing the potential applications of fermented legume proteins in functional foods and plant-based alternatives [13,14]. The effect of fermentation is heavily dependent on a number of factors, such as legume species, microbial strain [15], fermentation conditions and processing parameters. Therefore, the selection of suitable microorganisms and the optimization of fermentation processes are crucial to achieving consistent enhancements in nutritional quality, sensory properties, and technological efficiency. Industrial implementation also has to face problems concerning process standardization, fermentation time, energy consumption, downstream processing and quality control [16,17].

Although previous reviews have examined legume fermentation at length, particularly its effects on ANF reduction, digestibility, nutritional quality, and general functionality [18,19], the present review implements a more protein-centered and structure–function-oriented perspective. It examines how strain-specific proteolysis and molecular modifications alter protein composition and structure, and how these transformations affect solubility, emulsification, foaming, gelation, digestibility, and sensory properties [5,20].

Importantly, the review goes beyond the frequently reported benefits by critically addressing the limitations and trade-offs of fermentation, including excessive proteolysis, loss of desirable protein functionality, and variable outcomes among strains and substrates. It also considers processing and industrial challenges, such as prolonged fermentation, difficulties in process standardization and scale-up, and potential undesirable sensory effects [21,22]. Furthermore, it links these changes to practical food-system applications, discussing how fermented legumes or their protein ingredients can be incorporated into plant-based foods and other formulations to achieve targeted nutritional, techno-functional, and sensory properties [5,23].

Thus, the principal novelty of this review lies in integrating ANF mitigation, protein structural modification, structure–function relationships, fermentation limitations, sensory trade-offs, scalability, and food applications within a single mechanistic and application-oriented framework, rather than presenting fermentation solely as a strategy for improving nutritional quality.

Therefore, this review aims to provide a comprehensive overview of the role of fermentation in improving legume proteins. It focuses on the reduction of antinutritional factors, protein degradation and modification, improvements in protein digestibility and bioavailability, enhanced functional properties, and applications in food systems. Furthermore, it discusses the potential of combining fermentation with emerging processing technologies to develop high-quality, nutritious, and sustainable legume protein ingredients.

## 2. Literature Review Methodology

### 2.1. Literature Search and Selection

A broad literature search was conducted using the Web of Science (WoS) Core Collection. The search covered publications available from database inception to August 2026 and focused on the effects of fermentation on legume protein quality, antinutritional factors, digestibility, functionality, and food applications. The search strategy combined the following key terms using Boolean operators: (“legume protein” OR “legume-based protein”) AND (“fermentation” OR “fermented”) AND (“protein quality” OR “digestibility” OR “functionality”) AND (“antinutritional factor” OR “anti-nutritional factor”) AND (“food application” OR “food system). Relevant additional references were identified from the reference lists of selected publications when appropriate.

Studies were considered eligible when they investigated the fermentation of legume-based substrates and reported relevant effects on protein quality, digestibility, functionality, antinutritional factors, or food applications. Original research articles and relevant review papers published in English with accessible full texts were included. Studies unrelated to legume fermentation, lacking relevant information, or without accessible full texts were excluded.

The literature selection involved sequential screening of titles, abstracts, and full texts. During title screening, clearly irrelevant publications were excluded. Abstracts were subsequently assessed for relevance to legume proteins, fermentation, protein quality, antinutritional factors, and food systems. Full texts of potentially relevant publications were then evaluated against the predefined eligibility criteria. The selected literature was critically assessed and synthesized to provide an integrated overview of current knowledge, technological applications, and research gaps. As this was a narrative review, no formal systematic or scoping review protocol was followed.

References were managed using EndNote X7^®^ (Thomson Reuters, Toronto, ON, Canada).

### 2.2. Data Extraction

Information from the eligible publications was systematically extracted and cross-checked by the authors. The extracted data included first author, publication year, legume species, fermentation type and conditions, effects on protein quality and digestibility, changes in antinutritional factors, functional properties, and food-system applications. The extracted information was subsequently critically evaluated and synthesized to identify major findings, trends, limitations, and research gaps.

## 3. Antinutritional Factors in Legume Proteins

### 3.1. Classification of ANFs

The common antinutritional factors in legume proteins are phytates, tannins, protease inhibitors, lectins and saponins. Two reoccurring frameworks occur in the literature for the classification of these ANFs: composition and thermal stability (Table 1). Lectins and protease inhibitors are proteinaceous by nature, while non-protein ANFs include phytates, tannins, saponins and alkaloids [3]. In terms of thermal taxonomy, protease inhibitors and lectins are thermolabile, whereas phytate, tannins and saponins are heat stable ANFs [24]. Understanding the classification of ANFs could help with legume processing optimization for the manufacturing of more bio-accessible nutrients in legume-based foods. Antinutritional factors in legume seeds are measured by using a combination of wet-chemistry and bioassay methods. However, the most common method is spectrophotometric quantification for phytate, tannins, and saponins, while enzymatic inhibition assays are used for trypsin inhibitors and hemagglutination or proteomic methods are commonly employed for lectins.

### 3.2. Occurrence of Antinutritional Factors in Major Legumes

The concentration and composition of ANFs differ considerably among legume species, cultivars, environmental conditions, agronomic practices, seed maturity and post-harvest storage. This variability influences both the nutritional quality of legumes and processing interventions [2]. Consequently, understanding the distribution of ANFs among major edible legumes such as soybean and kidney beans is essential for selecting suitable processing technologies to maximize nutrient bioavailability while preserving beneficial phytochemicals. In many studies, soybean frequently showed high lectins, phytic acid, and saponins content. Among Canadian pulses, raw soybean had the highest lectins (692.8 HU/mg), phytic acid (22.91 mg/g), and oxalates [29]. Moreover, Gu et al. reported that soybean varieties differed in trypsin inhibitor and lectin content [30]. Soybean extracts also had especially high soyasaponin I amounts, 4–6 times more than lentils, peas, and beans [31]. Another widely consumed legume is chickpea which commonly contained tannins, phytic acid, trypsin inhibitors, saponins and raffinose family oligosaccharides (RFOs). Chickpea cultivars showed tannin at 0.07–0.22% and trypsin inhibitor at 9–31 mg/g [32]. Across cultivars, phytic acid ranged from 4.74–20.40 mg/g, and low-ANF genotypes were identified [33]. Furthermore, Kabuli chickpea (*Cicer arietinum*) also contained tannins (0.24–0.60 g TAE/100 g), phytic acid (17.61–29.72 mg/100 g), and RFOs (1.33–3.06 g/100 g) [34].

Kidney beans or common beans (*Phaseolus vulgaris* L.) often had high phytates, trypsin inhibitors, and lectins. Kidney bean had the highest phytates and trypsin inhibitor activity among chickpea, lentil, broad and kidney beans [35]. Moreover, common beans showed trypsin inhibitor levels of 21.8–138.5 TIU/g across landraces, with genotype and location effects [36]. The wide range observed among common bean landraces further demonstrates that comparisons among legumes should consider genotype rather than relying solely on species-level differences. In a comparison between faba beans (*Vicia faba* L.) and common beans, higher trypsin inhibitors activity was reported in common beans [37]. Lectins, trypsin inhibitors, saponins, phytate, vicine, and convicine were present in faba beans [38]. In Swedish faba beans cultivars, lectin ranged 0.8–3.2 HU/mg, trypsin inhibitor 1.2–23.1 TIU/mg, saponin 18–109 µg/g, phytate 112–1281 mg/100 g, vicine 403–7014 µg/g, and convicine 35.5–3121 µg/g [39].

Gultekin Subasi et al. reported that saponin levels differ by pea variety and that protein isolation leads to about a two-fold enrichment in saponins compared to flour across all tested varieties [40]. Phytate, tannins, and oxalates are present in lentil [41]. Furthermore, it was observed that ANFs differed among the cultivars of cowpea (*Vigna unguiculata*), although they remained concentrated in albumin and globulin fractions [42].

ANF profiles vary significantly among legumes, and highlight the need for legume-specific processing strategies to reduce ANFs and improve nutritional and techno-functional properties.

### 3.3. Mechanisms of Interference with Protein Digestibility and Mineral Bioavailability

Legumes are recognized as excellent sources of proteins, iron, zinc, calcium, magnesium, and other essential micronutrients, but the bio-accessibility of these nutrients is often limited due to complex physicochemical interactions between ANFs and macromolecules during digestion [3]. Various factors affect the extent of these interactions, including the category and concentration of ANFs, structural characteristics of proteins, processing techniques, and the physiological conditions of the digestive tract. Therefore, evaluation of the nutritional value of legumes requires both compositional evaluation and knowledge of the bio-availability of nutrients. Phytates chelate divalent minerals (Fe, Zn, Ca and Mg) and form insoluble phytate-mineral complexes [43]. Additionally, they bind proteins and digestive enzymes, leading to lower protein solubility and inhibition of proteolysis [3]. The trypsin and chymotrypsin inhibitors (serine-protease) hinder enzymatic activity by creating protein–protein interactions. Consequently, the enzyme action is repressed by blockage of the active site of the enzymes through the N- or C-terminus and exposed loop of protease inhibitors [44]. Trypsin inhibitors strongly inhibit trypsin and chymotrypsin, resulting in reduced digestion and absorption of dietary protein [45].

Lectins present in legumes bind to carbohydrate receptors on intestinal epithelial cells and nonspecifically interfere with secretion, nutrient absorption and mucosal function [46]. Tannins form complexes with proteins and mineral elements, rendering them unavailable for absorption and utilization. Such interactions can reduce protein digestibility and mineral accessibility and may consequently contribute to lower nutrient bioavailability [32]. Whereas, saponins form complexes with proteins and prevent protein digestion [47].

### 3.4. Health Implications of ANFs

ANFs have been reported with both positive and negative health implications. Therefore, ANFs cannot be labeled as simply “bad” compounds present in legumes. They are best understood as dose-dependent bioactive compounds that can impair nutrition when present at high levels or raw, but may also contribute to health benefits when present at lower levels or after processing [48,49].

Phytate typically lowers mineral absorption by binding metals, but it is also reported to have antioxidant and possible anticancer effects. Studies even found reduced colon and mammary cell proliferation with phytic acid supplementation [27]. Similarly, tannins can bind proteins and minerals and reduce digestibility, yet also contribute antioxidant and anti-inflammatory activity [48]. High levels of dietary trypsin inhibitors can cause substantial reductions in protein and amino acid digestibility [50]. Hypolipidemic and antitumoral properties of lectins and protease inhibitors have also been established. Reduced nutrient absorption and bloating is caused by saponins but they were also reported for their cholesterol-lowering and other bioactive properties [46,51].

## 4. Impact of Fermentation on Antinutritional Factors

Fermentation is an effective and sustainable bioprocessing method that is used to improve the nutritional quality of legumes by reducing ANFs. Unlike thermal treatment, which degrades thermolabile compounds, during fermentation, microbial metabolic activity produces an array of hydrolytic enzymes which degrade multiple ANFs and also improve digestibility [5]. The extent of these changes depends on the legume species and cultivar, microorganism and strain, fermentation type, substrate characteristics and preparation method, as well as processing conditions such as temperature, pH, fermentation time, moisture content and oxygen availability. Moreover, these factors can interact to determine microbial growth, enzyme activity and accessibility of ANFs within the legume matrix. Therefore, fermentation conditions should be optimized according to the target ANF and the characteristics of the substrate rather than assuming a uniform response across different legumes and fermentation systems [52].

### 4.1. Reduction of Phytates (Phytase Activity)

Phytates are salts of phytic acid (PA, Myo-inositol 1, 2, 3, 4, 5, 6-hexakis-dihydrogen phosphate, IP6) in its storage form. Fermentation is an important biological approach for reducing phytates by employing phytase (myo-inositol hexakisphosphate phosphohydrolase). Consequently, phytic acid is hydrolyzed into inorganic phosphate and myo-inositol phosphate derivative [8]. Fermentation of soybean, cowpea and ground bean with *Rhizopus oligosporus* caused 30.7, 32.6 and 29.1% phytic acid reduction, respectively [53]. In a study, soaking and germination followed by fermentation with *Lactiplantibacillus plantarum* 299v reduced phytates in maize flour by 85.6%, while fermentation alone led to 65.3% phytate reduction [6]. Moreover, co-culture fermentation is reported to have improved phytic acid reduction [7]. The differences in the extent of phytate reduction among these studies indicate that fermentation efficacy is strongly dependent on the processing system. The relatively similar reductions observed in soybean, cowpea and ground bean fermented with *R. oligosporus* may reflect the use of the same microorganism, whereas the substantially greater reduction reported when soaking and germination preceded fermentation may be associated with the combined effects of these pretreatments and fermentation. The higher reduction observed with co-culture fermentation further suggests that microbial composition and the associated phytase activities can influence phytate degradation. Furthermore, the extent of phytate degradation during fermentation is particularly influenced by microorganism or strain and its phytase activity, while pH, temperature, fermentation time and substrate preparation affect enzyme activity and the accessibility of phytate to microbial or endogenous phytases.

### 4.2. Degradation of Tannins and Polyphenols

Fermentation degrades tannins and can modify polyphenols in legumes. The degradation products of tannins can contribute to an increase in total phenol amounts post fermentation. Condensed tannins decreased by 30–50% during sourdough fermentation in Faba bean. While total phenols and antioxidant activity increased, implicating the microbial esterase activity and acidification in phenolic release [10]. Gelatinized flours of yellow and red lentils, white and black beans, chickpeas, and peas grains were subjected to fermentation with *Lactobacillus plantarum MRS1* and *Lactobacillus brevis MRS4*, resulting in improved degradation of phytic acid, tannins and raffinose than from fermentation alone [47]. For tannins, the extent of reduction depends on the microbial strain and its enzymatic capacity, together with fermentation duration, pH and substrate characteristics. Therefore, changes in total phenolic content should not necessarily be interpreted as direct evidence of tannin degradation because fermentation may also alter the extractability and chemical form of phenolic compounds.

### 4.3. Inactivation of Protease Inhibitors by Fermentation

Trypsin inhibitors are recognized as the main protease inhibitors across major legumes, including soybean, chickpea, kidney bean, faba bean, pea, cowpea, black gram, and lentils. LAB can produce extracellular and intracellular proteases that contribute to proteolysis during fermentation and may also contribute to the degradation or structural modification of proteinaceous trypsin inhibitors [9]. Moreover, acidification during fermentation can further alter the physicochemical environment and may affect the stability or activity of protease inhibitors [54]. Therefore, the reduction in trypsin inhibitor activity is likely to depend on the microorganism or strain, fermentation conditions and characteristics of the legume matrix. Mediterranean faba bean by *Lactobacillus plantarum DPPMAB24W* reported reductions in trypsin inhibitors ranging from 66.67% to 89.65% [55]. Moreover, beans fermented using whey showed superior quality features, including more decline in inhibitor activity, vibrant appearance and better sensory acceptability when compared to brine as a medium of fermentation [56]. Interestingly, trypsin inhibitors also reduced significantly when soybean, green pea and lentil flours were sonicated (2–4 min), followed by fermentation with *L. plantarum* [57]. The variation among these studies suggested that the reduction in trypsin inhibitor activity is influenced by both the fermentation system and pretreatment. The reported reduction in faba bean was obtained with a defined *L. plantarum* strain, whereas the greater reduction observed with whey compared with brine indicates that the fermentation medium can also affect inhibitor inactivation. Similarly, the additional sonication pretreatment used before fermentation of soybean, green pea and lentil suggests that disruption of the flour matrix may influence the accessibility of proteinaceous inhibitors to microbial or enzymatic action. Direct quantitative comparison among these studies should nevertheless be made cautiously because the legume species, microbial strain, substrate form and fermentation conditions differed. In addition to reducing protease inhibitor activity, fermentation may modify protein structure through microbial proteolysis, changes in protein–protein interactions and alteration of protein–ANF interactions. These changes can influence the accessibility of peptide bonds to digestive enzymes and consequently affect protein digestibility. However, the direction and magnitude of these changes are dependent on the legume matrix, microorganism and fermentation conditions [11].

### 4.4. Detoxification of Lectins and Other Compounds

**Figure 1:**
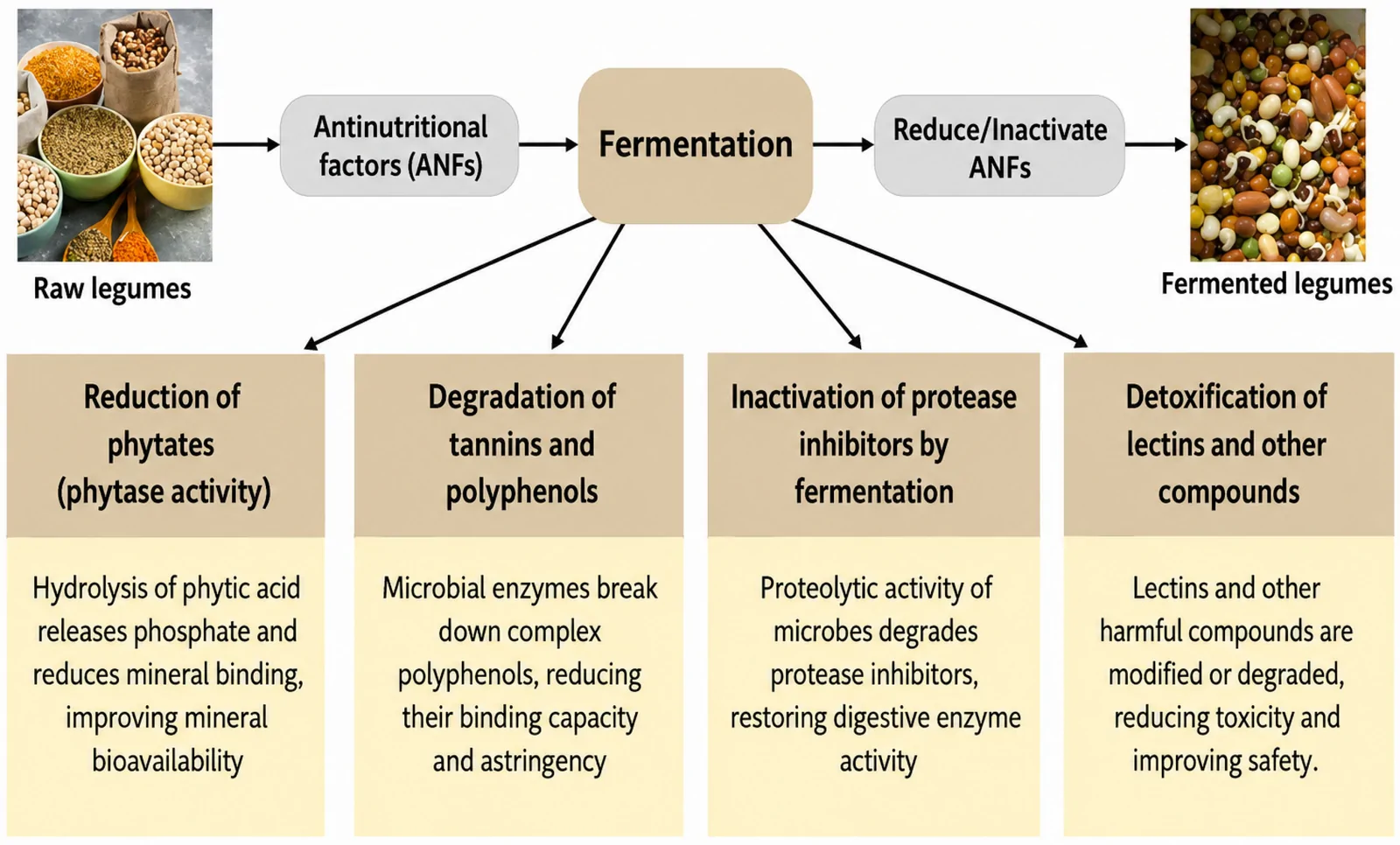
Effect of fermentation on antinutritional factors in legumes. Drawn in canva.

Lectins, due to their thermal instability, are often reduced to safer consumption levels both by cooking and fermentation [58]. Lactic fermentation using *Streptococcus thermophilus* 14,085 and *Bifidobacterium infantis* 14,603 at 37 °C for 24 h reduced saponins and phytates in soymilk [59]. Figure 1 illustrates the role of fermentation in ANFs in legumes. During fermentation, microbial enzymes degrade or inactivate these compounds through phytate hydrolysis, polyphenol breakdown, protease inhibitor degradation, and detoxification processes. These transformations can reduce the potential inhibitory effects of ANFs and may improve nutrient accessibility and protein digestibility; however, reduction in ANF content or activity does not by itself establish increased nutrient bioavailability. Demonstration of improved mineral bioavailability requires appropriate evidence of mineral accessibility, absorption or other validated nutritional outcomes. Figure 1 illustrates the effects of fermentation on antinutritional factors (ANFs) in legumes, highlighting the main ANFs affected by fermentation and the associated reduction mechanisms.

## 5. Protein Degradation and Modification During Fermentation

### 5.1. Proteolysis Mechanisms and Key Enzymes

Proteolysis is among the most important biochemical reactions that occur during legume fermentation. This protein degradation is due to the synergistic activity of endogenous seed proteases and microbial proteolytic enzymes that catalyze both the hydrolysis of legume proteins and the degradation of antinutritional compounds [60,61]. In legumes, the major storage proteins (7S vicilins and 11S legumins) constitute nearly 70–80% of the total protein fraction and represent the principal substrates of microbial proteolytic enzymes during fermentation [60]. These proteases play a crucial role in the conversion of storage proteins by cleaving them into smaller peptides and free amino acids (FAAs) able to be easily assimilated by microorganisms and to improve the nutritional quality of the substrate [12]. Furthermore, it was established that proteolysis not only enhances protein digestibility but also generates peptides with interesting biological activities [20]. It is also important to note that proteolytic mechanisms differ according to the fermenting microorganisms. Indeed, lactic acid bacteria (LAB) (e.g., *Lactobacillus plantarum*, *Lactobacillus casei* and *Pediococcus pentosaceus*) possess a specific proteolytic system including cell-envelope proteinases (CEPs) (encoded by *PrtP*, *PrtH*, *PrtR* or *PrtS* genes), peptide transport systems (oligopeptide permease “Opp”, dipeptide permease “Dpp” and di-/tripeptide transporter “DtpT”) and a wide range of intracellular peptidases such as aminopeptidases (PepN, PepC, and PepA), endopeptidases (PepO and PepF), dipeptidases (PepD and PepV), tripeptidases (PepT) and proline-specific peptidases (PepX, PepP, and PepQ) [62]. For a more comprehensive understanding of this mechanism, it could be underlined that extracellular CEPs firstly hydrolyze intact storage proteins into oligopeptides, which are subsequently transported into microbial cells, where intracellular peptidases generate short peptides and the FAAs required for microbial growth [60,62].

In alkaline fermentations of legumes such as those used for the production of some typical legume-based fermented foods like natto (Japan), kinema (India and Nepal), thua nao (Thailand) and dawadawa (West Africa), *Bacillus* species are frequently used, particularly *B. subtilis*, which exhibits a proteolytic activity stronger than LAB [61]. This microorganism is known to secrete diverse extracellular enzymes such as alkaline serine proteases (subtilisins), neutral metalloproteases and glutamyl endopeptidases able to rapidly hydrolyze globulins into low-molecular-weight peptides [12,61].

Furthermore, fungal fermentations like those involving *Rhizopus oligosporus* during tempeh (traditional Indonesian soybean-based fermented food) production, for example, contribute considerably to protein hydrolysis through the secretion of acid and neutral proteases, as well as amino- and carboxy-peptidases [63].

Besides microbial enzymes, endogenous legume proteases remain active during fermentation [60,61]. In fact, acidification, hydration and cell wall disruption facilitate the activation of vacuolar proteases, mainly cysteine and serine proteases, leading to a partial degradation of storage proteins before microbial proteases become dominant [61]. Effectively, the production of organic acids by fermentative microorganisms lowers the pH of the substrate, creating favorable conditions for endogenous and microbial enzyme activities while suppressing undesirable microorganisms [3,20]. Therefore, it could be perceived that the combined activity of plant and microbial enzymes results in a strong coordinated proteolytic process.

Moreover, it was established that proteolysis is influenced by fermentation parameters such as pH, temperature, fermentation duration, moisture content, oxygen availability, and microorganisms. For more details in this context, acid fermentation favors the activity of LAB peptidases under acidic conditions (pH 4.0–5.5), while alkaline fermentation promotes the production of extracellular proteases by *Bacillus* species at pH values above 7.0 [60]. Temperature also regulates enzyme synthesis, with optimal proteolytic activity generally occurring at between 30 °C and 40 °C, depending on the microbial species [20]. Regarding fermentation time, it was reported that its increase strongly enhances the degree of hydrolysis [64]. Added to that, adequate moisture content and oxygen availability (anaerobic or microaerophilic conditions) displayed pivotal roles in enhancing proteolysis through promoting microbial growth, substrate accessibility and enzyme diffusion [60]. Finally, selecting efficient starter cultures and optimizing the fermentation parameters generally result in more reproducible proteolytic patterns than spontaneous fermentation, thus contributing to improved nutritional, technological and organoleptic qualities in fermented legume-based foods [19,20,61]. Understanding these enzymatic mechanisms is therefore fundamental to designing fermentation strategies that maximize protein quality while generating useful protein changes and producing health-promoting bioactive peptides.

### 5.2. Changes in Protein Structure and Molecular Weight Distribution

Fermentation induces deep modifications in legume protein structure, affecting their transformation, molecular weight distribution, molecular interactions and solubility [60]. These transformations are driven by enzymatic proteolysis, acidification, and physicochemical changes, as well as the microbial metabolism occurring throughout fermentation [20]. They contribute to the conversion of compact storage proteins into smaller and more digestible protein fragments with improved nutritional value profiles and techno-functional properties [60]. In fact, the legume storage proteins displayed highly ordered secondary, tertiary and quaternary structures, stabilized by hydrogen bonds, hydrophobic interactions, electrostatic forces, and intermolecular disulfide bridges, such as in the case of legumins [14]. These structural characteristics confer remarkable stability, but also limit enzyme accessibility during gastrointestinal digestion [65]. During fermentation, microbial proteases progressively cleave peptide bonds leading to partial or significant unfolding of the native protein architecture [14]. Moreover, lactic acid fermentation associated with the decrease in pH could destabilize protein structures by disrupting electrostatic interactions and promoting protein swelling, thereby contributing to the exposure of buried cleavage sites to proteolytic enzymes [14]. In this line, several techniques attested these changes in protein conformational features. Effectively, spectroscopic analyses using circular dichroism (CD), Fourier-transform infrared spectroscopy (FTIR), and fluorescence spectroscopy have demonstrated a reduction in α-helical content associated with a relative increase in β-sheet structures, random coils, and flexible peptide regions during fermentation [60]. These architectural rearrangements increase molecular flexibility and surface hydrophobicity, but facilitate enzyme accessibility and water penetration into protein matrices [66]. In addition, disruption of intermolecular aggregates reduces steric hindrance, thereby improving protein susceptibility to digestive enzymes such as pepsin, trypsin, chymotrypsin and pancreatin [67].

On the other hand, it has been reported that one of the most significant consequences of fermentation is the redistribution of protein molecular weights. Indeed, the SDS-PAGE (sodium dodecyl sulfate-polyacrylamide gel electrophoresis) showed the gradual disappearance or weakening of high-molecular-weight globulin subunits (typically ranging from 40 to 70 kDa) along with the accumulation of low-molecular-weight peptides below 10–15 kDa [14,60]. Additionally, LC–MS (liquid chromatography–mass spectrometry) technique confirmed that extensive proteolysis shifts the peptide population toward shorter sequences composed of 2 to 20 AA residues [68]. This reduction in molecular weight is positively correlated with enhancements in protein digestibility, AA bioaccessibility, and biologically active peptide release [20].

Furthermore, the magnitude of structural modification depends strongly on the fermentation microorganism. In fact, LAB generally induce moderate and selective hydrolysis because of their intracellular peptidase systems, whereas *Bacillus subtilis* produces large quantities of extracellular alkaline proteases and metalloproteases able to extensively degrade the storage proteins into soluble peptides and FAAs [12,61]. Similarly, fermentations implicating fungi, particularly *Rhizopus oligosporus*, promote considerable degradation of protein matrices through the secretion of acid and neutral proteases, resulting in pronounced reductions in protein molecular weight and improved digestibility [63,69]. Consequently, alkaline fermentations (with *Bacillus* spp.) usually exhibit a greater degree of protein hydrolysis than acid fermentations (LAB and fungi), although the latter often preserve better sensory characteristics [61].

Beyond proteolytic degradation, fermentation also modifies the intermolecular interactions that govern protein functionality [14]. For further insight into this fact, it is worth noting that partial hydrolysis reduces protein aggregation while increasing molecular mobility, leading to enhanced solubility, emulsifying capacity, foaming properties, and water-holding capacity [70]. Acidification close to the isoelectric point initially decreases protein solubility; however, continued enzymatic hydrolysis generates smaller peptides possessing greater hydrophilicity and improved aqueous dispersibility [60]. These physicochemical changes are particularly valuable for developing innovative plant-based dairy alternatives, protein beverages, fermented legume ingredients and meat analogues with high functional performance.

In addition, advanced proteomic analyses have revealed that protein structural remodeling during fermentation is highly selective rather than random. Notably, proteases preferentially hydrolyze exposed peptide regions while preserving more resistant domains until later stages of fermentation [12]. Consequently, intermediate peptides accumulate transiently before undergoing further hydrolysis depending on fermentation duration and microbial enzymatic activity. This dynamic remodeling generates a heterogeneous peptide profile that ultimately determines both the nutritional and biological properties of fermented legumes [12].

Overall, the structural reorganization of legume proteins during fermentation represents a fundamental mechanism underlying improvements in protein quality [60]. The progressive conversion of compact globular proteins into small and structurally flexible peptides was recognized to offer several benefits: it (i) enhances digestibility, (ii) increases AA availability, (iii) improves functional properties, and (iv) creates molecular precursors for the generation of health-promoting bioactive peptides [12,20]. Therefore, understanding the relationship between protein structural changes and molecular weight distribution is crucial for optimizing fermentation processes in order to produce high-quality plant protein ingredients for future functional food applications.

### 5.3. Release of Peptides and Free Amino Acids (FAAs)

The release of peptides and FAAs constitutes one of the most significant biochemical consequences of legume fermentation and represents the direct outcome of microbial and endogenous proteolytic activities. As storage proteins are progressively hydrolyzed, complex globular structures are converted into oligopeptides, di-/tripeptides, and individual AAs that generate highly dynamic peptide profiles (such as hydrophobic residues, aromatic AAs, and proline-containing motifs…) and serve not only as essential nutrients for microbial growth but also as key contributors to the nutritional, biological, and sensory properties of fermented legumes [12,60]. The level of peptide and FAA accumulation depends on the degree of proteolysis, fermentation conditions, microbial strain, and intrinsic composition of the legume protein matrix [64]. Particularly, the accumulation of glutamic acid content is noteworthy in this context since it contributes to the characteristic umami flavor of fermented legume products such as tempeh, natto, kinema, and dawadawa [61]. Similarly, elevated concentrations of alanine, glycine, serine, and aspartic acid play an essential role in improving taste and overall flavor [64]. As mentioned above, proteolytic microorganisms exhibit highly coordinated enzyme systems that sequentially degrade proteins into smaller molecules. Specifically, extracellular proteases initially break down storage proteins into intermediate peptides, which are subsequently transported into microbial cells through ATP-binding cassette (ABC) peptide transporters. Then, these peptides undergo further hydrolysis by intracellular peptidases, leading to the release of FAAs that support microbial metabolism and protein biosynthesis [62]. Nevertheless, importantly, not all peptides are completely degraded; a substantial proportion accumulate in the fermented substrate and contribute to its improved nutritional and physiological functionality [12]. Furthermore, it has been reported that fermentation significantly increases concentrations of essential amino acids (EAAs) such as lysine, leucine, isoleucine, valine, phenylalanine and threonine, which are frequently limited in plant-based diets [71].

Concerning the microbial ability of peptides and FAA release, they differ across species. To be more precise, LAB generally produces diverse peptide mixtures due to their moderate proteolysis, characterized by the predominance of intracellular peptidase systems, whereas, *Bacillus* species (especially *Bacillus subtilis*) generate high levels of soluble peptides, amino nitrogen, and FAAs, resulting from extensive protein hydrolysis through the secretion of large quantities of extracellular alkaline proteases [12].

### 5.4. Formation of Bioactive Peptides

Among the most valuable biological outcomes of legume fermentation is the generation of bioactive peptides. These are specific protein fragments that display biological activities and physiological effects beyond their nutritional roles [20]. In addition, they are encrypted within the primary sequences of native storage proteins and remain biologically inactive until released through enzymatic hydrolysis during food processing, microbial fermentation or gastrointestinal digestion [72]. In this line, importantly, fermentation has been described as one of the most efficient and sustainable bioprocesses able to liberate these functional peptides, with their diverse and interesting biological activities [73].

The formation of bioactive peptides is mainly driven by the specificity of microbial proteolytic systems. In fact, during fermentation, the various proteinases progressively hydrolyze legume proteins into different shorter oligopeptides, which may exhibit biological activity, depending on their AA sequence, molecular size, hydrophobicity, and structural conformation [12,69].

Another point worth noting is that among the biological properties attributed to bioactive peptides derived from legume fermentation, antioxidant activity has been the most extensively investigated. This activity is strongly attributed to peptides containing hydrophobic AAs, sulfur-containing AAs (e.g., methionine and cysteine), aromatic residues (e.g., tyrosine, tryptophan, and phenylalanine), and histidine residues capable of stabilizing reactive oxygen species (ROS) [20,74,75]. Here, it has been demonstrated that fermentation significantly increases the antioxidant capacity of legume protein hydrolysates, thus contributing to the reduction in oxidative stress associated with chronic diseases. Another major group comprises angiotensin-converting enzyme (ACE)-inhibitory peptides, which have attracted considerable attention because of their potential antihypertensive effects, which offer a promising alternative to traditional medications [20,76]. Indeed, ACE catalyzes the conversion of angiotensin I into angiotensin II, a potent vasoconstrictor involved in blood pressure regulation [77]. Short peptides released during fermentation can competitively inhibit ACE activity, reducing angiotensin II formation and consequently promoting vasodilation [76]. Then, peptides containing hydrophobic AAs or proline residues at the C-terminal position generally exhibit the highest inhibitory potency [76]. Fermented bean, soybean, pea, chickpea, and lentil proteins have all been reported to contain ACE-inhibitory peptides generated through microbial proteolysis [12,20,72]. Hence, these peptides generated by fermentation may have a potential antihypertensive effect; however, their direct ability to exert clinically relevant blood pressure lowering remains to be established in vivo through appropriate animal models and, ultimately, well-controlled human intervention studies.

Recent research has also highlighted the ability of fermented legume peptides to inhibit dipeptidyl peptidase IV (DPP-IV), an enzyme involved in the degradation of incretin hormones such as glucagon-like peptide-1 (GLP-1) [78]. The observed DPP-IV inhibitory activity suggests that these peptides may interfere with an enzymatic pathway relevant to glucose homeostasis. Mechanistically, inhibition of DPP-IV could contribute to the preservation of endogenous incretin activity, thereby potentially prolonging GLP-1-mediated stimulation of glucose-dependent insulin secretion and improving postprandial glycemic regulation, suggesting potential relevance to the dietary modulation of glycemic responses. Moreover, several fermentation-derived peptides have been observed to exhibit α-glucosidase- and α-amylase-inhibitory activities, thus contributing to the modulation of postprandial glucose responses [78]. Therefore, all of these physiological effects should be considered as indicative of their potential antidiabetic relevance and could not be directly inferred from in vitro assays or be evidence of improved glycemic control or therapeutic efficacy in the management of type 2 diabetes, as biological activities in vivo depend on several factors (peptide stability, peptide concentration, interaction with food, ability to reach molecular targets, etc.). Accordingly, further in vivo studies are required to determine whether these in vitro findings translate into clinically relevant effects.

Added to that, numerous peptides generated during protein legume fermentation displayed antimicrobial, immunomodulatory and cardiometabolic properties [20,79]. Antimicrobial activity may be explained by the fact that cationic peptides enriched in lysine and arginine could interact with negatively charged microbial membranes, which increases membrane permeability and ultimately causes microbial cell death [80]. For the immunomodulatory effect of some bioactive peptides, it could be correlated to their capacity in regulating innate and adaptive immune responses by modulating cytokine secretion, macrophage activation, and inflammatory signaling pathways. Then, anti-inflammatory peptides have been shown to suppress the activation of nuclear factor-kappa B (NF-κB), reduce the production of pro-inflammatory mediators, and attenuate oxidative damage in cellular models [79]. Hence, further clinical assays on humans remain necessary to confirm these biological effects.

The biological activities of fermentation-derived peptides depend on several structural determinants (e.g., peptide length), amino acid composition, charge distribution, hydrophobicity, and resistance to gastrointestinal digestion [80]. In general, small peptides, particularly dipeptides and tripeptides, exhibit the greatest bioavailability because they can resist digestive degradation and be transported across the intestinal epithelium through peptide transporter 1 (PEPT1) [68]. However, not all peptides generated during fermentation reach systemic circulation intact; many exert local effects within the gastrointestinal tract or are further cleaved into smaller active fragments during digestion [14,69]. Consequently, evaluating peptide bioactivity requires integrating peptidomic characterization with simulated gastrointestinal digestion and cellular or in vivo validation. Despite the growing evidence supporting the health-promoting properties of fermentation-derived peptides, several challenges remain to be addressed. Effectively, beyond the encouraging results of in vivo human studies with less reliance on in vitro experiments, microbial strain selection, substrate composition, and processing parameters strongly influence peptide profiles, highlighting the need for standardized fermentation protocols aimed to produce reproducible and efficient bioactive peptides. Future research combining controlled fermentation, advanced techniques, and human-based clinical validation will undoubtedly strengthen the role of fermented legumes as sustainable sources of naturally occurring bioactive peptides for the prevention and management of non-communicable diseases.

### 5.5. Balance Between Degradation and Nutritional Enhancement

Protein degradation during fermentation represents a highly dynamic process in which the extent of proteolysis ultimately determines whether fermentation results in nutritional improvement or undesirable quality deterioration. That is why controlling fermentation constitutes a delicate strategy to maintain or enhance the nutritional value of legume proteins. Firstly, moderate protein hydrolysis has consistently been associated with significant improvements in protein nutritional quality [60]. To support this point, Adebo et al., Du et al. and Carbonaro et al., recognized that partial proteolysis disrupts the compact quaternary structure of legume storage proteins, increasing their accessibility to digestive enzymes and thereby enhancing protein digestibility [19,20,67]. Simultaneously, fermentation reduces the concentration of antinutritional compounds such as trypsin inhibitors, tannins, lectins, and phytates, which otherwise impair protein digestion and mineral bioaccessibility [61]. Hence, the combined effects of protein unfolding, enzymatic hydrolysis, and antinutritional factor degradation substantially promote bioactive peptide generation and improve both AA utilization and protein bioaccessibility [19,60].

Other studies attested that the improvement in protein quality through controlled proteolysis could be assessed by nutritional indices such as increases in in vitro protein digestibility (IVPD), digestible indispensable AA scores (DIAAS), and protein digestibility-corrected AA scores (PDCAAS) following fermentation of some legumes (soybean, chickpea, lentil, and pea) [14,71]. These observed enhancements result not only from improved enzymatic accessibility but also from the partial degradation of protein–polyphenol and protein–phytate complexes that otherwise limit gastrointestinal digestion [60]. Consequently, it is clear that fermentation contributes to a more efficient use of FAAs as well as EAAs, which are important for muscle protein synthesis and metabolic health [71]. On the other hand, it has been reported that controlled proteolysis also plays a central role in improving the techno-functional properties of legume proteins, making them more suitable for various food applications. In fact, legume protein hydrolysis could increase molecular flexibility, surface hydrophobicity, and protein solubility, leading to enhanced emulsifying, foaming, water-holding, and gelling capacities which are valuable for the development of novel and more sustainable food formulations (e.g., high-protein functional foods, plant-based dairy alternatives, fermented beverages, and meat analogues) [70,81]. Although protein degradation is essential for providing nutritional advantages such as generating bioactive peptides, increasing AA bioaccessibility, enhancing digestibility, and reducing antinutritional factors, over-hydrolysis can generate peptide populations that are too small to stabilize emulsions or foams, and form cohesive gel networks, thereby reducing functional properties, processing performance and product stability [82]. Also, uncontrolled proteolysis may negatively affect protein quality by promoting undesirable sensory attributes (in terms of flavors, textures and tastes) of fermented legume-based foods [61]. For instance, several studies showed that extensive degradation of storage proteins result in the excessive accumulation of FAAs and hydrophobic AA (e.g., leucine, isoleucine, phenylalanine, valine, and tyrosine) which could contribute to bitter taste and undesirable flavor profiles [20,70]. Moreover, excessive AA catabolism by microorganisms, resulting from their use as nitrogen and carbon sources for growth, may (i) produce ammonia, biogenic amines, sulfur-containing volatile compounds, and branched-chain aldehydes, (ii) potentially reduce the availability of certain EAAs, (iii) decrease the biological value of the protein fraction, and (iv) impair both sensory acceptability and food safety if fermentation is prolonged and not adequately controlled [12,71]. Therefore, the nutritional success of legume fermentation highly depends on achieving an optimal balance between controlled proteolysis and nutritional enhancement by the preservation of protein integrity. This balance is affected by multiple fermentation processing variables, as detailed above, including microbial strain selection, inoculum concentration, temperature, pH, moisture and oxygen availabilities, substrate composition and fermentation time [20,60]. Indeed, starter cultures exhibiting moderate but highly specific proteolytic systems generally produce more desirable nutritional outcomes (as already mentioned) than spontaneous fermentations, which often display greater variability in microbial succession and protease expression [19,20]. Therefore, mixed starter cultures combining well-selected LAB with particular *Bacillus* or fungal species have recently attracted considerable interest because they enable complementary enzymatic activities for efficient peptide production while minimizing excessive protein degradation and undesirable metabolite formation [12,61].

Additionally, modulating proteolytic activity according to predefined nutritional objectives could represent promising advances in food biotechnology for optimizing legume protein fermentation processes. Indeed, integrated approaches combining genomics, proteomics, peptidomics, metabolomics, and biological systems may allow deep characterization of microbial protease expression, peptide release kinetics, and AA metabolism throughout fermentation [60,64]. These multi-omics technologies largely facilitate the rational selection of microbial strains capable of maximizing digestibility and bioactive peptide production while minimizing nutrient losses and sensory defects. Furthermore, artificial intelligence (AI)-assisted peptide predictive models are emerging as valuable tools for optimizing fermentation parameters and predicting proteolytic outcomes based on microbial metabolic networks [83].

Table 2 reviews and associates fermentation conditions (e.g., microorganism, temperature, time, pH, and substrate) and highlights their effects on protein quality, digestibility, and ANFs in different legume-based substrates. It emphasizes how fermentation can enhance protein utilization while reducing compounds such as phytates, tannins, protease inhibitors, lectins, and oligosaccharides.

## 6. Effects on Protein Digestibility and Bioavailability

### 6.1. Improvements in In Vitro and In Vivo Digestibility

Legumes are a sustainable and economical source of plant-based protein. However, their nutritional value is often limited by their lower digestibility compared to animal proteins. This low digestibility results primarily from the compact structure of 7S vicilins and 11S legumins, their association with antinutritional compounds, and the presence of complex cellular matrices. This complexity of the plant matrix limits access by digestive enzymes. Fermentation is now recognized as one of the most effective biotechnological strategies for improving the physiological utilization of legume proteins [60].

During fermentation, microorganisms produce a wide range of proteases, peptidases, and hydrolytic enzymes. These enzymes are capable of partially degrading native proteins into low-molecular-weight peptides and free amino acids. This proteolysis leads to significant structural changes, including protein unfolding, reductions in β-sheet structures, and exposure of hydrophobic groups buried within the protein matrix. These changes significantly increase the accessibility of cleavage sites to gastrointestinal enzymes such as pepsin, trypsin, and chymotrypsin [89]. These structural changes can be characterized using complementary analytical techniques. SDS-PAGE offers qualitative information on the disappearance of high-molecular-weight protein subunits and the formation of lower-molecular-weight fragments following fermentation. Size-exclusion chromatography (SEC-HPLC) can provide a more quantitative assessment of changes in molecular-weight distribution. Fourier-transform infrared spectroscopy (FTIR) and circular dichroism can further be used to monitor changes in protein secondary structure, while fluorescence-based measurements can provide information on changes in tertiary structure and exposure of hydrophobic residues. The combination of these techniques with digestibility assays is particularly useful for establishing whether fermentation-induced improvements in protein digestibility are associated with protein unfolding, partial hydrolysis, disruption of aggregates, or other structural modifications. The evaluation of these improvements currently relies primarily on standardized in vitro digestion methods. The INFOGEST protocol has become the international standard for simulating the physiological conditions of human digestion and comparing the nutritional quality of dietary proteins. Using this approach, Sousa et al. developed a methodology to estimate the Digestible Indispensable Amino Acid Score (DIAAS) based on in vitro digestion data [90]. The authors observed an excellent correlation between the DIAAS values obtained using the INFOGEST method and those measured in vivo, with a correlation coefficient as high as 0.96. This result demonstrated the relevance of this approach for evaluating the quality of plant-based and fermented proteins. This advancement is particularly significant because DIAAS is now considered by the FAO to be a more reliable indicator than the Protein Digestibility-Corrected Amino Acid Score (PDCAAS) for assessing the nutritional value of proteins. Although data on the DIAAS of fermented legumes remains limited, the available results suggest that fermentation-induced changes promote better digestibility of essential amino acids and could thus improve these nutritional indices. The assessment of fermentation-induced improvements in protein quality requires the use of complementary analytical endpoints rather than a single digestibility measurement. In vitro protein digestibility (IVPD) can be estimated from the disappearance of protein nitrogen, the release of primary amino groups, the degree of hydrolysis, or the recovery of individual amino acids after simulated gastrointestinal digestion. DIAAS is particularly relevant because improvements in total protein digestibility do not necessarily indicate proportional improvements in the availability of all indispensable amino acids. Therefore, amino acid composition should be determined before and after fermentation, while amino acid-specific digestibility should preferably be assessed after simulated or in vivo gastrointestinal digestion. The Protein Digestibility-Corrected Amino Acid Score (PDCAAS) integrates the concentration of the limiting amino acid with protein digestibility, whereas the DIAAS considers the true ileal digestibility of individual indispensable amino acids, and therefore provides a more informative estimate of protein quality. For fermented legumes, combining IVPD with amino acid profiling and DIAAS is particularly useful for determining whether fermentation improves not only protein hydrolysis but also the nutritional availability of limiting indispensable amino acids [90]. In vitro studies conducted on fermented legume proteins have largely confirmed these observations. Liu et al. investigated the structural changes in chickpea proteins during lactic acid fermentation carried out with *Lactobacillus plantarum*. The results showed that fermentation caused partial hydrolysis of storage proteins, a decrease in β-sheet structures, and increased exposure of hydrophobic groups [89]. These changes significantly improved protein hydrolysis during simulated gastrointestinal digestion. These results demonstrated a notable increase in vitro protein digestibility. The authors concluded that the fermentation-induced reorganization of the protein structure was a major mechanism explaining the improved enzymatic accessibility and, consequently, the nutritional value of the fermented proteins. Kim et al. evaluated the effect of a treatment combining enzymatic hydrolysis pea proteins (*Pisum sativum*) and lactic acid fermentation carried out using *Lactobacillus plantarum CKDHC 0801* and *Lactobacillus brevis KCCM 11509* [91]. The authors observed a reduction of nearly 99% in the average molecular weight of the proteins and a decrease of more than 53% in β-sheet structures. These changes led to a 22.5% increase in in vitro digestibility compared to the unfermented protein isolate. The study also showed a 38.4% improvement in uptake potential in a Caco-2 intestinal cell model. This result indicates that the peptides generated by fermentation were more readily absorbed in the intestine [79]. The authors concluded that fermentation not only improves protein digestibility but also helps increase their bioaccessibility after digestion. Beyond in vitro models, several in vivo studies confirm the nutritional benefits associated with fermentation. Animal models, particularly those using pigs, are frequently used to assess true ileal protein digestibility and determine DIAAS values [79]. These studies have generally shown that fermentation promotes better digestive utilization of proteins through the pre-hydrolysis of macromolecules, the reduction in antinutritional factors, and improved access of digestive enzymes to protein substrates. Fermented proteins also generate a greater quantity of low-molecular-weight peptides and free amino acids during digestion, which promotes their intestinal absorption. However, despite the evidence accumulated in animal models, in vivo studies specifically focused on fermented legumes remain relatively few. Comparisons between studies remain challenging due to the heterogeneity of fermentation processes, microbial strains used, and methods for assessing digestibility [92].

Data from human studies are even more limited, but the available results suggest that fermentation may improve the metabolic availability of essential amino acids. Pre-hydrolyzed or fermented proteins appear to be digested more rapidly and to produce higher plasma concentrations of essential amino acids after ingestion. Furthermore, studies using Caco-2-type cellular models have shown that peptides generated by fermentation exhibit greater transport capacity through the intestinal epithelium. Thus, currently available data support the notion that fermentation simultaneously improves gastrointestinal digestibility and the release of essential amino acids, their bioaccessibility and eventually their bioavailability.

### 6.2. Interaction Between ANF Reduction and Protein Accessibility

The low digestibility of legume proteins is not solely due to their intrinsic structure, but also to the presence of various antinutritional factors (ANFs). These factors can limit the access of digestive enzymes to protein substrates. Among the main ANFs are phytates, protease inhibitors, tannins, lectins, and certain indigestible oligosaccharides. These compounds can interact directly with dietary proteins or with digestive enzymes. This interaction can reduce the bioavailability of amino acids and the overall nutritional value of legumes. Fermentation is now recognized as one of the most effective technologies for reducing these compounds, while simultaneously improving the accessibility and physiological utilization of proteins [5].

The analytical determination of ANFs requires consideration of both their concentration and their biological activity. Phytate, for example, can be determined using colorimetric or precipitation-based assays, whereas HPLC and ion-exchange chromatographic methods provide greater specificity and can distinguish phytic acid (InsP6) from lower phosphorylated inositol phosphates (InsP3–InsP5). This distinction is particularly significant during fermentation because microbial phytases progressively hydrolyze phytate into lower inositol phosphates rather than necessarily eliminating phytate completely. Consequently, reporting only total phytate concentration may not fully describe the biochemical transformation occurring during fermentation [93,94].

Phytic acid (myo-inositol hexakisphosphate) is one of the most abundant ANFs in legumes. Its strong chelating capacity allows it to form insoluble complexes with proteins and essential minerals such as iron, zinc, calcium, and magnesium. These interactions not only reduce mineral bioavailability but also limit the access of digestive enzymes to the associated proteins. During fermentation, many lactic acid bacteria, yeasts, and fungi produce phytases capable of gradually hydrolyzing the phosphate groups of phytic acid. These enzymes cause the dissociation of protein–phytate complexes and the release of nutrients previously trapped in the food matrix. Several studies have shown that lactic fermentation of legumes such as peas, fava beans, soybeans, or chickpeas has led to a substantial reduction in phytate content. This fermentation has thus promoted a concomitant improvement in protein digestibility and mineral bioavailability [5]. From an analytical perspective, the extent of phytate degradation should ideally be assessed by combining total phytate determination with chromatographic profiling of individual inositol phosphate species. Such an approach is particularly relevant for fermented legumes because partial dephosphorylation may substantially modify the mineral-binding capacity of phytate even when measurable amounts of InsP6 remain. Therefore, the nutritional significance of fermentation-induced phytate reduction depends not only on the percentage decrease in total phytate but also on the degree of dephosphorylation and the resulting profile of lower inositol phosphates [93].

Trypsin and chymotrypsin inhibitors represent another major group of ANFs that affect the nutritional quality of plant proteins. These inhibitory proteins, primarily from the Kunitz and Bowman–Birk families, bind to pancreatic enzymes and reduce their hydrolytic activity in the small intestine. This inhibition leads to reduced protein digestion and decreased amino acid absorption. Fermentation contributes to their degradation through the combined action of microbial proteases and the acidification of the environment. Lactic acid bacteria, as well as certain species of Bacillus and filamentous fungi, are capable of hydrolyzing these inhibitors into low-molecular-weight peptides, thereby reducing their biological activity. This reduction in protease inhibitors is considered one of the key mechanisms explaining the improved digestibility observed in fermented legumes [95]. Because the nutritional relevance of protease inhibitors is primarily related to their inhibitory activity, functional assays are preferable to measurements based solely on inhibitor concentration. Trypsin inhibitor activity is commonly determined using enzymatic assays based on the residual activity of trypsin toward a chromogenic substrate such as Nα-benzoyl-DL-arginine-p-nitroanilide (BAPNA). An international collaborative study demonstrated the reproducibility of an improved standardized procedure for determining trypsin inhibitor activity in legumes, cereals, and related products [96]. Chymotrypsin inhibitor activity can be assessed using analogous enzymatic approaches, although greater methodological variability remains among published protocols. Accordingly, residual inhibitory activity should preferably be reported together with concentration-based measurements when evaluating the effect of fermentation.

Condensed tannins also constitute a significant barrier to the digestive utilization of proteins. These polyphenols have a strong affinity for dietary proteins. They form stable complexes through hydrogen bonds and hydrophobic interactions. The formation of these complexes reduces protein solubility and limits their enzymatic hydrolysis in the gastrointestinal tract. During fermentation, several microorganisms produce oxidative and hydrolytic enzymes capable of modifying the structure of phenolic compounds and reducing their ability to bind to proteins. Furthermore, acidification of the medium often promotes the partial dissociation of protein–tannin complexes, and increases the accessibility of proteins to digestive enzymes [5]. The analytical determination of tannins also requires methodological caution because the response of colorimetric assays depends on tannin structure and extraction conditions. Condensed tannins are frequently estimated using vanillin–HCl or acid-butanol assays, whereas chromatographic approaches provide greater chemical resolution. Recent methodological guidance emphasizes that differences in solvent composition, extraction time, temperature, calibration standards, and reaction conditions can substantially influence the measured tannin content. Therefore, comparisons of tannin reduction among fermented legume studies should consider the analytical method and reporting basis used rather than relying exclusively on the reported percentage reduction [97].

Lectins represent another category of ANFs frequently found in legumes. These glycoproteins can bind to carbohydrates on the surface of intestinal cells and disrupt the normal function of digestive mucosa. Their presence can impair nutrient absorption and reduce digestive efficiency. Although heat treatment remains the most effective method for lectin inactivation, several studies indicate that fermentation has also contributed to their partial degradation through the proteolytic activity of fermentative microorganisms. This hydrolysis reduces their biological activity and helps to improve the nutritional quality of the fermented proteins [5]. Oligosaccharides of the raffinose family, primarily raffinose, stachyose, and verbascose, are traditionally associated with gastrointestinal discomfort linked to the consumption of legumes. Although they do not directly affect protein digestibility, their presence influences the perception and acceptability of legume-based products. Fermentative microorganisms produce α-galactosidases. This enzyme is capable of hydrolyzing these oligosaccharides into simple sugars that can be used as a carbon source. The reduction of these compounds not only improves digestive tolerance but also alters the structure of the food matrix. This can indirectly facilitate the accessibility of proteins to digestive enzymes [5]. Lectins are commonly assessed using hemagglutination assays, which provide a functional estimate of residual lectin activity, although immunochemical or chromatographic methods may provide greater specificity. In contrast, raffinose-family oligosaccharides, including raffinose, stachyose, and verbascose, are generally quantified using chromatographic methods such as HPLC with refractive-index or evaporative light-scattering detection. These approaches allow individual oligosaccharides to be distinguished and are therefore useful for assessing microbial α-galactosidase activity during fermentation. More broadly, the use of standardized extraction procedures, reference standards, reporting units, and validated analytical methods is essential for meaningful comparison of ANF reduction across fermentation studies [94,98].

The improved protein accessibility was related to a combination of complementary mechanisms. First, microbial enzymes directly degrade several ANFs, and can reduce their inhibitory effect on digestive processes. Second, the breakdown of protein–ANF complexes releases cleavage sites previously inaccessible to gastrointestinal enzymes. Third, fermentation profoundly alters the organization of the food matrix through partial solubilization of cellular components, hydrolysis of macromolecules, and reorganization of protein networks. Finally, prior microbial proteolysis increases the specific surface area available for enzymatic attack and promotes the subsequent release of peptides and amino acids during digestion. These synergistic effects explain why the improvements in digestibility observed after fermentation are often greater than those achieved by reducing antinutritional factors alone. From this perspective, fermentation should be viewed not only as a strategy for detoxifying legumes but also as a biotransformation tool capable of simultaneously optimizing the digestibility, accessibility, and bioavailability of plant proteins.

From a methodological perspective, the relationship between ANF reduction and protein accessibility can be strengthened by combining ANF measurements with indicators of protein structural modification. Protein solubility assays, SDS-PAGE, SEC-HPLC, FTIR, and fluorescence spectroscopy can provide complementary information on protein extraction, molecular-weight distribution, secondary structure, and tertiary structural changes. When these measurements are combined with IVPD or INFOGEST-derived digestibility, they can help distinguish the contribution of ANF degradation from that of microbial proteolysis, protein unfolding, aggregation disruption, or food-matrix disintegration. Thus, a decrease in ANF concentration should not be interpreted as direct evidence of improved protein quality unless it is accompanied by functional evidence of increased enzymatic accessibility or digestibility.

### 6.3. Gastrointestinal Fate of Fermented Proteins

In the stomach, pepsin is the primary enzyme responsible for the initial hydrolysis of dietary proteins. Pre-fermented proteins generally have a more open and partially hydrolyzed structure, facilitating pepsin access to peptide cleavage sites. This increased susceptibility results in faster gastric hydrolysis and early production of low-molecular-weight peptides. In the case of fermented pea proteins, Kim et al. observed that an increase in the cellular bioavailability of digestion products demonstrated that the modifications induced prior to ingestion persist throughout the digestive process [91]. The products generated during gastrointestinal digestion can be characterized using complementary electrophoretic, chromatographic, and mass-spectrometric approaches. SDS-PAGE and size-exclusion chromatography provide information on the disappearance of intact proteins and the formation of lower-molecular-weight peptides, whereas LC–MS/MS-based peptidomics enables the identification and sequencing of digestion-derived peptides. Amino acid analysis can further quantify the release of individual amino acids following intestinal digestion. These approaches are particularly valuable for fermented legumes because microbial proteolysis occurring before ingestion may alter the peptide profile subsequently generated by pepsin, trypsin, chymotrypsin, and intestinal peptidases. Consequently, the gastrointestinal fate of fermented proteins should be evaluated not only by overall protein disappearance but also by changes in peptide size distribution, peptide sequence, and amino acid release.

After passing through the stomach, the remaining proteins and peptides are subjected to the combined action of intestinal pancreatic enzymes, primarily trypsin, chymotrypsin, and carboxypeptidases. These enzymes continue to hydrolyze the proteins into shorter peptides, dipeptides, tripeptides, and free amino acids. Since fermented proteins are already partially degraded, their intestinal hydrolysis is generally more efficient than that of native proteins. This enhanced digestion promotes increased release of essential amino acids while increasing the amount of biologically active peptides available in the intestinal lumen. Recent studies reported that fermentation is one of the most effective processes for stimulating the generation of functional peptides during gastrointestinal digestion [99].

Among the compounds generated during the digestion of fermented proteins, bioactive peptides are attracting growing interest due to their potential health benefits. Several studies have shown that fermented pea, soybean, fava bean, and other legume proteins release peptides with antioxidant, antihypertensive, anti-inflammatory, and immunomodulatory activities. Angiotensin-converting enzyme (ACE)-inhibiting peptides are among the most extensively studied. Jakubczyk et al. demonstrated that simulated gastrointestinal digestion of pea proteins fermented by *Lactobacillus plantarum* led to the release of peptides exhibiting strong ACE-inhibitory activity [100]. These results suggested a potential antihypertensive effect following consumption [100]. More recently, peptidomics studies have confirmed that pea protein hydrolysates contain low-molecular-weight peptides associated with antioxidant and ACE-inhibitory activities. These investigations reinforce the interest in fermented proteins as a source of functional bioactive compounds.

Intestinal absorption is a critical step for the expression of the biological effects of these peptides. Dipeptides and tripeptides resulting from digestion are primarily transported across the intestinal epithelium by the oligopeptide transporter PEPT1, located on the apical membranes of enterocytes. This system enables the efficient absorption of numerous bioactive peptides before they are completely hydrolyzed into free amino acids. Available data suggest that fermentation promotes the production of peptides of a size compatible with this transport mechanism, and increase their potential bioavailability. Beyond nutrient absorption, fermented proteins and their digestion products can also interact with the gut microbiota. This area of research is currently developing rapidly and represents one of the most promising avenues in precision nutrition. Unabsorbed peptides and protein fractions that reach the colon can be metabolized by gut microorganisms. Fermented foods have been linked, in particular, to an increase in certain beneficial bacterial populations and to enhanced production of microbial metabolites that promote gut health. Among these, short-chain fatty acids (SCFAs), such as acetate, propionate, and butyrate, play an essential role in maintaining the integrity of the intestinal barrier, immune regulation, and energy metabolism of the host. The interactions between fermented plant proteins, bioactive peptides, and the gut microbiota thus represent an additional mechanism that may explain the physiological benefits associated with the consumption of fermented legumes [101]. Future studies should increasingly combine standardized INFOGEST digestion with dynamic gastrointestinal models, LC–MS/MS peptidomics, and metabolomics to provide a more comprehensive assessment of the fate of fermented proteins. Such integrated approaches could help determine whether structural and compositional changes induced during fermentation persist throughout gastrointestinal digestion and ultimately translate into greater release and absorption of indispensable amino acids or bioactive peptides.

**Figure 2:**
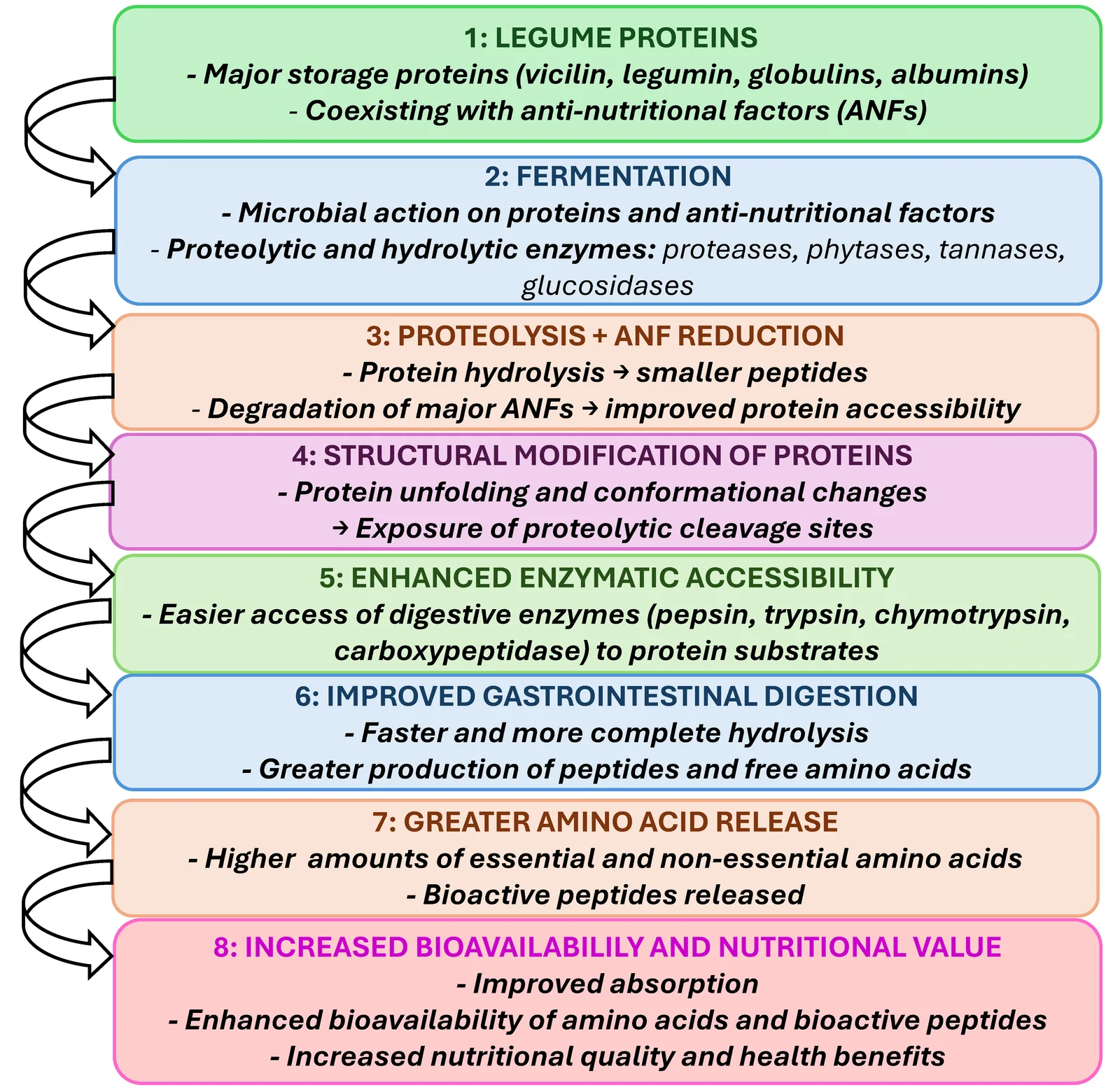
Mechanisms by which fermentation improves protein digestibility and bioavailability in legumes.

Despite the progress made, several scientific challenges remain. Most current knowledge is still based on static digestion models such as INFOGEST, which do not fully replicate the physiological dynamics of the human gastrointestinal tract. Future research should rely more heavily on dynamic digestion models capable of incorporating temporal variations in digestive conditions as well as interactions with the microbiota. Furthermore, approaches such as peptidomics, digestive proteomics, and metabolomics now offer new possibilities for precisely identifying the peptides generated during digestion and tracking their metabolic fate. Combined with emerging strategies in nutritional precision fermentation, these technologies should enable the development of fermented plant proteins specifically designed to generate peptides that target particular physiological functions, and pave the way for a new generation of personalized functional foods. Mechanisms by which fermentation recovers legume protein digestibility through microbial proteolysis, reduction of antinutritional factors, release of bioactive peptides, and enhanced accessibility of amino acids during digestion and their bioavailability are summarized in Figure 2.

## 7. Limitations and Negative Effects of Legume Protein Fermentation

If proteolysis is inadequately achieved, fermentation of legume proteins can reduce functional properties, produce undesirable sensory attributes, and decrease nutritional quality [102,103,104]. The intensity of these properties depends on strain selection, fermentation duration, and substrate concentration, with outcomes varying widely across legume types and processing conditions [60,105,106].

### 7.1. Deterioration of Techno-Functional Properties

Throughout fermentation, excessive proteolysis methodically challenges the techno-functional properties that make legume proteins valuable as food ingredients. According to García Arteaga (2021), fermentation of pea protein isolate with six lactic acid bacteria strains for 24 and 48 h could reduce emulsifying capacity from 550 mL/g in the unfermented control to as low as 180 mL/g, and reduce protein solubility [102]. Similarly, fermentation of lupin protein isolates decreased solubility at pH 7 from 64% to below 41%, although foaming activity and emulsifying capacity remained comparable to the unfermented control [107]. Submerged fermentation of lentil protein isolate with *Aspergillus niger*, *Aspergillus oryzae*, and *Lactobacillus plantarum* preserved foaming capacity but reduced foam and emulsion stabilizing properties and decreased water-holding capacity across all fermented samples compared to a control [103]. In another study, Stone et al., (2024) stated that solid-state fermentation of chickpea, green lentil, and faba bean protein isolates by the same microbial strains reduced water-holding capacity and emulsion stability after 48 h [105]. In addition, solid-state fermentation of fava bean flour with *A. oryzae* and *Rhizopus oligosporus* generated protein aggregates that increased particle size and reduced solubility, while emulsions prepared with the fermented ingredients separated faster [108]. Lactic acid fermentation during pea protein isolation decreased powder solubility by 20%, although water absorption and foaming capacity increased twofold [109].

### 7.2. Nutritional Limitations and Reduced Digestibility

Solid-state fermentation of green lentil, faba, and chickpea bean protein isolates could decrease protein digestibility for all pulses, which hypothesized to result from the increased phenolic content caused by microbial metabolism [105]. In addition, fermentation can enhance antinutritional compounds, viz. saponins and phenolics, which inhibit proteolytic digestion [103,105]. In faba bean tempeh, trypsin inhibitor activity was not condensed by fermentation, continuing as a persistent antinutritional factor [110]. Similarly, according to Gautheron et al. (2024), fava bean fermentation with filamentous fungi could provoke a reduction in arginine, an essential amino acid, possibly due to acidic destabilization [108]. In addition, gelatinization preceding fermentation could negatively touch the antioxidant activity of legume flours despite enhancing other nutritional parameters [47]. Commonly, lactic acid fermentation is less operative than enzymatic hydrolysis in releasing polypeptides and minimizing allergenicity. Therefore, fermentation alone may be insufficient to produce hypoallergenic ingredients. As an illustration, in fermented pea protein, compact intensity of allergenic protein fractions was attributed to reduced protein solubility instead of genuine proteolytic degradation of the strains [102].

### 7.3. Sensory Deterioration from Excessive Proteolysis

While controlled fermentation, could reduce beany and green off-flavors in legume proteins, unwarranted proteolysis produces new undesirable sensory features. Fermentation of pea protein isolate for 48 h augmented cheesy aroma and acidic and salty tastes, and fermentation with *Lactobacillus fermentum* produced a fecal aroma that was the least preferred by the sensory panel [102]. According to Gao et al., (2024) [111], additional degradation of amino acids through fermentation presented a positive correlation with off-flavors, chiefly the enrichment of 3-methylbutanoic acid in fermented soybean proteins. In addition, alkaline fermentation with *Bacillus* spp. generated ammonia through protein degradation, producing atypical pungent odors that reduce sensory quality [104]. On the other hand, a high microbial activity or excessive fermentation promotes high ammonia concentrations that give off unpleasant odors [104].

Alternatively, the fermentation of legume proteins could provoke textural degradation. For example, excessive proteolysis triggers weaker or less stable protein matrices, reducing the structural functionality required in food systems [104]. In addition, proteolytic activity increasingly decreases chewiness and hardness over fermentation time as the protein structure is modified.

## 8. Applications in Food Systems

### 8.1. Legume-Based Spreads

Specific starter cultures and fermentation conditions (temperature and pH) need to be investigated for plant-based applications such as cheese alternatives, hence providing optimal chemical and sensory properties [112]. The functionality of legume-based spread was compared for production from protein concentrates such as brown pea (AB), yellow pea (AY), and faba bean (AF) using different fermentation patterns and starter cultures. Legume-based spreads were fermented using a starter culture from Danisco, Copenhagen, Denmark, (containing *Streptococcus thermophilus* and *Lactobacillus delbrueckii* subsp. *bulgaricus*) for plant-based products; and a starter culture (*Lactococcus lactic* subsp. *cremoris*, *Leuc. pseudomesenteroides*, *Lactococcus lactis* subsp. *Lactis biovar diacetylactis*, *Lactococcus lactis* subsp. *Lactis*, *Leuconostoc mesenteroides*) for dairy products from Chr. Hansen Holding, Denmark. AY-based spreads presented better sets of characteristics, as reported by Illarionova et al. [113].

### 8.2. Mung Bean Snacks

Mung bean (*Vigna radiata* L.) is a plant-based protein source, containing approximately 27% protein, with a comparable amino acid profile to soybean and kidney bean, with 59–65% carbohydrates, 1–1.5% fat, 3.5–4.5% dietary fiber, and 4.5–5.5% ash on a dry weight basis [114,115]. It can be employed in extruded mung bean chips, baked legume-based snacks, high-protein crackers, grissini, and gluten-free puffed snacks, as well as vegan and clean-label products [116]. They investigated the impact of rosemary and thyme-based coatings combined with oven, microwave, and air-fry roasting on the physicochemical, bioactive, and volatile properties of a high-protein mung bean snack and found that the air-fried, rosemary-coated samples had the highest protein content (25.96%).

### 8.3. Fermented Legume-Based Foods

Lentils exhibit versatile functional properties, including water- and oil-holding capacity, emulsification, solubility, foaming, and gelation, which support their use as stabilizers, gelling agents, or thickeners [117]. Fermentation constitutes a promising alternative technology to effectively reduce ANFs while simultaneously enhancing bioactive components, improving digestibility, and positively modifying the nutritional and sensory attributes of pulses and derived food products [3]. The probiotic properties of *Lactiplantibacillus plantarum 299v*) have been well discussed [118]. This microorganism can enhance the functional properties of foods through its ability to ferment plant-based substrates (including legume-derived raw materials) and improve their nutritional and bioactive profiles [119,120]. Various *L. plantarum* strains have been used in the fermentation of legume substrates, including lentils [57,121,122], with solid-state fermentation being the most frequently applied approach. Skrzypczak et al. investigated the impact of fermentation with probiotic strain *L. plantarum 299v* on the techno-functional characteristics and selected ANFs of lentil flour [15]. They found that fermentation significantly reduced ANFs, while maintaining or enhancing the techno-functional integrity of lentil flour.

### 8.4. Plant-Based Meat Analogues (PBMAs)

Plant-based meat analogues incorporating meat-like characteristics constitute suitable substitutes that confer benefits in terms of both health and ecological impact, while addressing the limitations of real meat. Meat analogues provide a variety of options for individuals with specific dietary preferences such as vegans, vegetarians, and flexitarians due to their substantial physicochemical characteristics [123,124], including lab-grown, tissue-engineered meat from cell cultures and plant-based meat or mycoprotein-based meat from ascomycetes or basidiomycetes to improve the juiciness, chewiness, and appearance of analogues [125]. Plant-based proteins derived from legumes, oilseeds, cereals, and pseudocereals could replace meat, as they form a nutritionally balanced diet with sustainable and well-preserved functionality [123]. Texturized vegetable proteins (TVPs) comprise plant-based analogues closely imitating the myofibrillar assembly of meat, thereby contributing to organoleptic structures [126]. Mycoprotein obtained from the mycelia of filamentous fungi has also been used in meat analogues [127]. The fibrous texture of meat analogues can be enhanced by hydrocolloids, specifically when subjected to high moisture extrusion (HME) and high-temperature shear cell (HTSC) processing [128]. Production of fibrillar-oriented structures arises from coagulation, texturization, and extrusion of proteins [129]. Sher et al. provided an updated information about novel protein sources from legumes, oilseeds, cereals, pseudocereals, mushrooms, fungi, and algae species, also addressing the role of key ingredients such as binding agents, colorants, flavorants, chemical constituents and processing technologies, to improve the functionality of PBMAs [130].

## 9. Challenges and Future Perspectives

Fermentation is a promising approach to improving the nutritional quality, digestibility and techno-functional properties of legume proteins. However, several challenges need to be solved before fermentation can be implemented on an industrial scale. The heterogeneity of microbial ecosystems, variability of legume substrates, and the lack of standardized processing conditions limit the reproducibility and scalability of fermented legume protein ingredients.

### 9.1. Standardization of Fermentation Processes

One of the main challenges in the development of fermented legume proteins is the absence of standardized fermentation protocols. The outcome of fermentation is largely influenced by parameters such as the selection of microbial strain, inoculum size, substrate composition, moisture content, temperature, pH, fermentation time and oxygen availability [131]. These parameters have a major effect on protein hydrolysis, peptide release, degradation of antinutritional factors (ANFs), metabolite production and final sensory properties [132]. Traditional spontaneous fermentation offers complex microbial ecosystems that may generate diverse metabolites and enhance nutritional quality, but the uncontrolled succession of microorganisms may introduce variability between batches and potential safety issues. Controlled fermentation with selected starter cultures improves reproducibility but reduces microbial diversity and metabolic complexity [133]. Therefore, the development of standard fermentation conditions adapted to particular legume species remains essential. Also important for the assessment of fermentation efficiency are standardized analytical methods. To establish quality benchmarks for fermented legume ingredients, systematic monitoring of parameters such as degree of hydrolysis (DH), amino acid availability, reduction in phytate and tannins, peptide bioactivity, improvement in digestibility and microbial metabolites should be performed [4]. Therefore, future studies should focus on developing prediction models relating fermentation parameters to nutritional and functional outcomes [134]. The application of artificial intelligence (AI), machine learning and process analytical technologies can enable real-time monitoring and optimization of fermentation processes [135].

### 9.2. Optimization of Microbial Consortia

The second major challenge for fermented legume proteins is the selection and optimization of microbial communities. Different microorganisms possess complementary enzymatic activities that influence protein modification and ANF degradation. Lactic acid bacteria (LAB), *Bacillus* spp., yeasts and fungi have been shown to produce proteases, phytases, tannase and other enzymes associated with the improvement of protein nutritional quality [136,137]. For example, LAB fermentation can lower phytate levels through microbial phytase activity and Bacillus fermentation can lead to significant proteolysis and peptide formation by extracellular proteases [138]. However, single-strain fermentation may lack sufficient metabolic diversity compared to traditional mixed fermentations. The construction of optimized microbial consortia with complementary functions is therefore an emerging strategy. Recent advances, such as metagenomics, metabolomics and proteomics, offer novel opportunities to identify functional microorganisms and to understand microbial interactions during fermentation. These approaches can give rise to the development of optimized starter cultures, which simultaneously enhance the nutritional quality, sensory properties and functional performance of legume proteins [139].

### 9.3. Integration with Emerging Protein Technologies

Traditional extraction approaches commonly yield protein isolates with low solubility, poor digestibility, and unfavorable sensory properties [1]. Therefore, the combination of fermentation and innovative protein extraction and modification technologies is a promising strategy to overcome current limitations [5]. Fermentation can change the structures of proteins, make them more accessible to enzymes and produce functional peptides, which can make them more useful in sophisticated food applications [140]. However, some techno-functional limitations can be overcome by technologies such as ultrasound-assisted extraction, high-pressure processing, enzymatic hydrolysis, extrusion and microencapsulation. These technologies often do not completely remove thermostable antinutritional factors such as phytates and tannins [1], but the combination of these methods with fermentation improves the digestibility, sensorial quality and release of bioactive compounds [141,142]. Specifically, extrusion-assisted fermentation may improve the production of plant-based meat analogues through improving protein interactions, flavor development and textural qualities [142,143]. High moisture extrusion fermentation in pea-based sausages reduced pea-like odor, enhanced texture and added yeast-like and umami notes, and has demonstrated a clean-label technique to develop taste and structure in situ [142].

New technologies present fresh chances to enhance fermented legume proteins even more. Certain proteins, peptides, amino acids, and other functional molecules with improved nutritional and technological qualities can be produced using precision fermentation and microbial biotechnology [143]. Furthermore, combining fermentation with other processing techniques like protein fractionation, enzymatic hydrolysis, extrusion-based texturization, and microencapsulation can enhance the functionality, digestibility, and sensory quality of proteins while increasing their use in plant-based foods [141,143,144].

### 9.4. Sustainability and Economic Aspects

Fermentation presents significant opportunities for creating sustainable legume-based protein systems in addition to improving nutrition. Legumes are important crops for sustainable food production because they fix soil nitrogen and use fewer agricultural inputs than animal protein sources. By turning low-value biomass into useful ingredients, fermentation can increase the use of underutilized legumes, boost nutritional value, and decrease food waste [3,145]. Nevertheless, economic challenges associated with industrial-scale fermentation remain, such as equipment purchases, energy usage, fermentation duration, downstream processing, and quality assurance [17]. Although fermentation can improve nutritional value, additional processing steps may increase environmental impacts if energy-intensive technologies are employed. Therefore, life-cycle assessment (LCA) approaches are required to evaluate the overall sustainability of fermented protein production systems [146]. Furthermore, consumer acceptance remains a key factor influencing market expansion. Fermented plant-based proteins’ sensory attributes, public familiarity with this type of food, and clean-label expectations, will influence their adoption [146].

## 10. Conclusions

Fermentation has emerged as one of the most promising and sustainable strategies for enhancing the nutritional and functional quality of legume proteins. By reducing major antinutritional factors, including phytates, tannins, protease inhibitors and lectins, fermentation improves protein digestibility, mineral bioavailability, and overall nutritional value. Additionally, microbial proteolysis encourages structural changes in proteins, releasing free amino acids and bioactive peptides with possible health benefits. Furthermore, fermentation enhances the techno-functional properties of legume proteins, such as their solubility, emulsifying ability, foaming ability, and gelation, which make it easier to incorporate them into novel food products like fermented foods, plant-based snacks, spreads, and meat substitutes.

Despite these developments, the type of legume, microbial strain, and processing conditions continue to have a significant impact on fermentation results, underscoring the necessity for standardized procedures and optimized microbial consortia. Enhancing protein functionality and expanding plant-based protein applications may be possible by combining fermentation with cutting-edge technologies including enzymatic hydrolysis, extrusion, protein separation, and precision fermentation.

## Figures and Tables

**Table 1:** Antinutritional factors in legumes and their properties.

ANFs	Composition	Stability	Typical Role in Legumes	Reference
Phytates (phytic acid)	Non-protein	Heat stable	Chelate minerals and reduce mineral absorption; can also bind proteins and digestive enzymes, lowering protein solubility and proteolysis	[25]
Tannins	Phenolic/non-protein	Heat stable	Precipitate proteins, reduce digestibility, and lower amino acid availability; also reduce palatability	[26]
Protease inhibitors	Proteinaceous	Heat labile	Inhibit trypsin/chymotrypsin and reduce protein digestibility; major legume families include Kunitz and Bowman–Birk inhibitors.	[27]
Lectins	Proteinaceous	Heat labile	Carbohydrate-binding proteins that can agglutinate cells and interfere with nutrient absorption; some are toxic or growth inhibitory, though severity varies by species.	[28]
Saponins	Non-protein	Heat stable	Can interact with bile acids, reduce absorption of some lipids, and cause bitter taste and foaming.	[3]

**Table 2:** Comparative overview of fermentation conditions and their effects on protein quality and anti-nutritional factors in legume-based substrates.

Legume/ Substrate	Fermentation Type	Microorganism	Fermentation Conditions	Effects on Proteins/Amino Acids	Effects on Anti-Nutritional Factors	Other Relevant Effects	Reference
Faba bean flour	Lactic acid fermentation	*Lactiplantibacillus plantarum* VTT E-133328	30 °C, 48 h; flour/water dough	- Increase in free amino acids, particularly essential AA; - Increase in in-vitro protein digestibility	- Decrease in vicine and convicine; - Decrease in trypsin inhibitor activity and condensed tannins in protein-rich fraction	- Decrease of starch hydrolysis index; - Increase of GABA (Gamma-Aminobutyric Acid)	[84]
Faba bean flour	Controlled lactic acid fermentation	*L. plantarum* DPPMAB24W	30 °C, 48 h; 50:50 flour/water (*w*/*v*); 7,3 log CFU/g	- Increase in protein bioavailability and free AA; - Protein digestibility increased from approximately 42–69% to ≈90% in Mediterranean accessions	- Strong reduction in trypsin inhibitors, condensed tannins, α-galactosides and vicine/convicine	- Strong acidification; - Increase in β-glucosidase activity	[55]
Red/yellow lentil, white/black bean, chickpea, pea flours	Lactic fermentation, with/without gelatinization	*L. plantarum* MRS1 + *L. brevis* MRS4	30 °C, 24 h; raw or previously gelatinized flours	- Increase in free AA; - Increase in in-vitro protein digestibility	- Decrease in phytic acid, condensed tannins, raffinose and trypsin inhibitory activity	- Decrease in starch hydrolysis index; - Increase in radical-scavenging activity; - Strong reduction in overall anti-nutritional factors	[47]
Black chickpea flour	Lactic acid fermentation	*L. plantarum* T0A10	30 °C, 24 h; Dough yield 285; microbial starter ≈ 7.2 log CFU/g initially	- Increase in in vitro protein digestibility from ≈80 to 91%; - Increase in most free AA and GABA	- Reduction in raffinose, condensed tannins and saponins by approximately 50%; - Decrease in trypsin inhibitor activity	- Increase in resistant starch; - Increase in antioxidant activity	[85]
Soybean, lentil and green pea flours	Submerged lactic fermentation	*L. plantarum* and *Pediococcus acidilactici*	37 °C; 72 h; 1:8 substrate/water (*w*/*v*); pH 6.5; 10^8^ CFU/mL; 200 rpm	- Changes in AA profile; - No consistent increase in in vitro protein digestibility	- Decrease in total phenolics and trypsin inhibitory activity; - Decrease in phytic acid	- Effect depended strongly on substrate and pretreatment (sonication/precooking)	[57]
Lentil flour	Solid-state fungal fermentation	*Pleurotus ostreatus*	30 °C, 48 h	- Increase in protein content by approximately 23%; - Increase in digested protein fraction	- Reduction in anti-nutritional factors associated with improved protein accessibility	- Increase in polyphenols and antioxidant activity; - Increase in resistant starch; - Decrease in starch hydrolysis	[86]
Pea, faba bean and lentil	LAB fermentation+ propionic acid bacteria	Mixed LAB starters including *P. freudenreichii*	Pretreatment: soaking, crushing and boiling; 30 °C, 3 days anaerobic + 1 day aerobic at room temperature; dry matter 13–19%	- Nutritional quality improved indirectly through degradation of anti-nutritional factors; - Increase in protein bioaccessibility	- Significant decrease in galacto-oligosaccharides; - Degradation of vicine/convicine in faba bean; - Moderate effect on tannins	- Production of vitamin B12 by LAB/PAB consortia; - Marked changes in phenolic profile	[87]
Faba bean protein concentrate	Submerged bacterial fermentation	*L. fermentum*, *L. argentoratensis*, *B. subtilis*, *B. velezensis*	8% *w*/*v* faba bean protein concentrate; 30 °C; 200 rpm; 10^5^ CFU/mL; 8–72 h	*- Bacillus* spp. showed strong proteolysis and increase in essential free AA and γ-glutamyl peptides	*- L. argentoratensis* and *B. velezensis* significantly decrease vicine/convicine	- Significant decrease in hexanal and other beany aldehydes; - Strain-specific aroma modulation	[61]
Common bean, lentil and chickpea	Solid-state fermentation (SSF)	*Bacillus subtilis*	-12 h or 48 h; duration-dependent SSF	- 12 h improved AA digestibility and DIAAS (Digestible Indispensable Amino Acid Score,) in chickpea; - 48 h generally reduced AA digestibility, especially in lentil	-Early decrease in anti-nutritional factors during fermentation	- Prolonged fermentation can become detrimental to protein quality; - DIAAS remained <75%	[88]

## Data Availability

No new data were created or analyzed in this study. Data sharing is not applicable.
